# TGN1412: From Discovery to Disaster

**DOI:** 10.4103/0975-1483.66810

**Published:** 2010

**Authors:** H Attarwala

**Affiliations:** *Department of Pharmaceutical Sciences, School of Pharmacy, Northeastern University, Boston, MA, USA*

**Keywords:** Clinical trials, Fialuridine, TGN1412

## Abstract

After a drug is confirmed as safe and efficacious in preclinical studies, it is tested in healthy human volunteers for first in man trials. In 2006, a phase I clinical study was conducted for a CD28 superagonist antibody TGN1412 in six human volunteers. After very first infusion of a dose 500 times smaller than that found safe in animal studies, all six human volunteers faced life-threatening conditions involving multiorgan failure for which they were moved to intensive care unit. After this particular incident, a lot was changed over how first in man trials are approved by regulatory authorities and the way clinical trials are conducted. This review primarily deals with preclinical studies conducted by TeGenero, results of which encouraged them to test the antibody on human subjects, reasons why this drug failed in human trial and aftermath of this drug trial. In addition, another drug—Fialuridine which failed in phase 2 clinical trial leading to death of five human subjects is briefly reviewed.

## INTRODUCTION

CD28 superagonist antibodies can cause activation and proliferation of regulatory T cells regardless of signal received by T-cell receptor. Regulatory CD4^+^CD25^+^ T cells play an important role in prevention of autoimmune diseases.[1] Activation of regulatory T cells by antigens is controlled by co-stimulatory signal from antigen presenting cells, mainly dendritic cells (DC) where antigen is presented by MHC complex of DC to T cells via T-cell receptor. This along with co-stimulation of CD28 receptor by CD80 or CD86 ligand on DC is required for T-cell activation.[2] *In vitro* it was possible to stimulate T cells by the use of combination of antibodies against T-cell receptor and CD28 receptor. Monoclonal anti-CD28 antibody such as TGN1412 was capable of activating T cells by binding to CD28 receptor irrespective of T-cell receptor activation and hence it was termed as a CD28 superagonist. Superagonistic activity of these antibodies was shown to be as a result of their binding to C”D loop of CD28 receptor in contrast to other CD28 antibodies which bind to a site close to binding site of natural ligands. Since activation of regulatory T cells can be useful for the treatment of a variety of autoimmune diseases and cancer, they were investigated for their therapeutic potential in different animal models for their superagonist activity.[3] One such antibody TGN1412 by TeGenero underwent rigorous preclinical investigation prior to its approval for clinical trials. TGN1412 could cause *ex vivo* expansion of T cells in the absence of additional stimuli from T-cell receptor. In preclinical studies, well-tolerated expansion of T cells was observed without any measurable proinflammatory reaction. Moreover, TGN1412 also demonstrated its therapeutic potential for use in autoimmune disease because of its capability of activating regulatory T cells. Thus, depending upon the condition of the immune system TGN1412 was thought to be useful for disease related to low numbers of activated T such as B-cell lymphoma or for treatment of autoimmune diseases such as rhematoid arthritis. When this antibody was tested in humans, it was immediately withdrawn from phase 1 clinical trials and volunteers had to be taken to intensive care unit 8 h after drug infusion due to multiorgan failure.[4]

## DEVELOPMENT OF TGN1412

After identification of CD28 antibodies capable of activating T cells along with signal from T-cell receptors, studies were conducted to evaluate T-cell activation potential of these CD28 antibodies. Large number of mouse hybridomas were isolated and investigated for functional activity through CD28. It was found that one category of these antibodies was capable of activating T cells irrespective of signal received from T-cell receptor. They were named as CD28 superagonists. These antibodies did not differ in antibody class or the binding avidity for the CD28 receptor but differed in the epitope-binding site. Conventional CD28 antibody-binding site was at the top of CD28 molecule where the natural CD28 ligands bind, while the CD28 superagonist required an intact CD28 C”D loop for its binding.[2] Toward further development of this class of antibodies, TeGenero started with screening of several mouse monoclonal CD28 superagonist antibodies. From these studies, TGN1412, a genetically engineered humanized anti-CD28 antibody was produced by transferring complement-determining regions from variable regions of heavy and light chains of monoclonal anti-mouse CD28 antibody 5.11A1 into human heavy and light chain variable antibody construct. Huminized heavy and light variable regions were then combined with IgG4γ and κ chain coding human gene. A mouse antibody used in humans may have toxicity problems related to immunogenicity and problems related to effective functioning of antibody. To avoid these problems, the above humanized antibody TGN1412 was constructed.[2]

## *IN VITRO* EVALUATIONS OF TGN1412 IN HUMAN AND NON-HUMAN CELLS

Specificity of TGN1412 to CD28 was evaluated by flow cytometry and Biacore analysis. These assays showed specificity of TGN1412 for CD28 receptor and that TGN1412 did not cross react with other closely related molecular targets such as Cytotoxic T-lymphocyte-antigen-4 and inducible co-stimulator. *In vitro* studies for cross reactivity of TGN1412 with CD28 expressed on T cells of rodents and non-human primates revealed that TGN1412 had low-binding affinity for rodent CD 28 whereas the same was high in case of T cells from for CD 28 to T cells derived from cynomolgus monkey and rhesus monkey. Determination of sequence homology of C”D loop of CD28 of humans and rhesus revealed difference of one amino acid while that in marmoset monkey revealed difference of two out of six amino acids. In case of rodents, the C”D loop sequence homology with humans was very low. When incubated with different subsets of T cells obtained from healthy donars, only TGN1412 but not conventional CD28 antibody was able to cause rapid proliferation of T cells in the absence of stimuli from T-cell receptor. These results showed that TGN1412 had superagonistic activity for T cells obtained from healthy donars and that they could specifically react with CD28 receptor having sequence homology with human CD28 receptor.[2]

## *IN VIVO* STUDIES

Prior to use of TGN1412 different antibody variants were used for preclinical studies. All these studies demonstrated that these superagonist are safe and efficacious (Investigation brochure, 2005). These encouraging results demonstrated high possibility for the use of this superagonist for the treatment of different T-cell deficiency syndromes like auto-immune diseases and B-cell lymphoma. To further evaluate its efficacy, humanized antibody as described above was engineered from 5.11A1 mouse human CD28 antibody. Selection of proper non-human primate model was an important issue for testing further safety and efficacy of this antibody. Toward this end, cynomolgus and rhesus monkeys were chosen because the CD28 receptor in these species and humans have similar affinity for TGN1412[5] because of 100% sequence homology of extracellular domain of CD28 receptor.[5] Moreover, Fc receptors and their motifs responsible for signal transduction in these species are highly conserved in human species hence similar antibody affinities and response can be expected. On the basis of this hypothesis, it was decided that results obtained from pharmacokinetic and pharmacodynamic studies in these closely related species would most closely predict fate of drug response when tested in humans. A repeat dose study for toxicokinetic evaluation of TGN1412 was conducted. In this study, doses ranging from 5 to 50 mg/kg were administered. Plasma half-life of TGN1412 was found to be 8 h which was as expected for a large protein molecule like an antibody. Despite four increasing repeated doses of TGN1412 resulting in four plasma peaks concentrations of TGN1412, only one peak for increase in T-cell number was observed. This was because extent of expansion of T cells by TGN1412 is highly dependent on availability of T cells and saturation kinetics of CD28 co-stimulator receptor. After these studies, toxicological studies using rhesus and cynomolgus monkeys were conducted. Rodent species were not considered appropriate because of difference in binding affinities of TGN1412 at the C”D loop of CD28 receptor. A repeat dose pilot study was conducted in cynomolgus and rhesus monkey. In this study, an increasing dose of TGN1412 starting from 5 to 50 mg/mL was administered. Dose as high as 50 mg/mL was well tolerated and no adverse reactions such as systemic immune system disregulation or hypersensitive reactions were observed. In addition, no signs of toxicity were observed in any of the physiological systems including cardiovascular system, respiratory system, or central nervous system. On the basis of these results, no observed drug effect level was considered to be 50 mg/kg. For additional toxicity studies, rat anti-CD28 antibody jj316 or TGN1112 (IgI variant of TGN1412) were used for toxicological studies in relevant species. Expected pharmacodynamic effect of TGN1412, that is elevation in levels of CD4 ^+^ and CD8 ^+^ was observed after 13 days of initial dosing. Levels of IL-2, IL-6, and IL-5 were moderately increased in serum in animals treated with TGN1412. However, from these studies there was no indication or sign of any clinical manifestations of first dose cytokine release syndrome in any of the CD28 superagonist antibody-treated animals since elevation of cytokine levels was observed only for a week at 5 mg/kg dose of TGN1412. In addition, there was no signal from any of the animals treated with any dose of superagonist indicating symptoms of anaphylactic shock or development of autoimmune disease, or systemic immune suppression. In addition to these studies, tissue cross-reactivity studies were performed where distribution of lymphocytes was observed by lymphocyte staining. These studies revealed a consistent tissue staining in lymphoid tissue as expected demonstrating target-tissue specificity of CD28 superagonist. In addition, studies for immunogenicity of TGN1412 were performed on primate model. Anti-TGN1412 antibody titers were observed in all animals, which were thought to be as a consequence of the humanized antibody being used in primate model.[1–5] Hence, TGN1412 proved to be safe and efficacious and passed a variety of conventional preclinical safety tests such as *in vitro* tests on human white blood cells and preclinical tests in non-human primates which bagged TeGenero approvals from UK and German regulatory authorities for first in man phase 1 trial for this new therapeutic agent with an unusual mode of action.[6]

## CLINICAL DEVELOPMENT OF TGN1412

After getting approval from regulatory authorities, phase 1 trials were conducted. The main aim was to establish safe human dose which can be further be used for subsequent drug trials. For this purpose, it was decided to conduct the trials on healthy human volunteers because disease free subjects have comparable CD28 receptors as in case of rhematioid arthritis or B-cell lymphoma. Also, immunological safety was expected to be more in healthy subjects compared to those with pre-existing disease. In addition, healthy subjects would not only exclude effects of other medications administered to diseased patients, but also exclude the effects of functional activation or dysfunctionalization of T cells as a result of prior diseased condition.[2]

## DOSE CALCULATION

Since TGN1412 showed specificity toward CD28 receptor expressed on human and non-human primate T cells, safe dose calculated from preclinical studies in non-human primate model was considered of suitable relevance for calculation of first in human dose. Various tests for expected pharmacological activity of TGN1412 and unexpected toxicological effects of TGN1412 were conducted in non-human primates cynomolgus and rhesus monkeys. These tests demonstrated a dose of 50 mg/kg administered for four consecutive weeks to be safe[21] On the basis of the repeat dose toxicity studies in cynomolgus monkeys, no observed adverse effectt level (NOAEL) was considered to be 50 mg/kg per week for not less than four consecutive weeks. Considering FDA guidelines, “Minimal Anticipated Biological Effect Level” (MABEL) approach and the Safety Criteria for the safe first dose, a dose of 0.1 mg/kg was decided to be administered to healthy volunteers in a double-blind randomized placebo-controlled phase I clinical trial conducted.

## THE FIRST DOSE DISASTER

After collection of this large amount of preclinical data, when TGN1412 was administered to six healthy human volunteers in phase 1 clinical trial conducted by Paraxel for TeGenero at Northwick hospital in London, UK, minutes after the first infusion of humanized CD28 superagonist TGN1412, all patients started suffering from severe adverse reaction resulting from rapid release of cytokines by activated T cells.[7] Table 1 lists some of the important lessons from TGN1412 trial failure.

**Table 1:** Summary of learning points from the TGN1412 phase I study

TGN1412 study problem	Detail	Learning Point
Interpretation of preclinical (primate) studies	Low-level cytokine release in primate studies should have promoted more caution	Minor but potentially important effects in preclinical studies should raise caution in crossing the species barrier
Use of human *in vitro* studies	Insufficient *in-vitro* human studies were performed	*In vitro* studies on human material as close as possible to the target tissue can be important.
Choice of starting dose	Subtle difference between primate and human target ligand may explain marked difference in potency – the calculation of an initial dose based on a fraction of predicted ‘no adverse effect level’ proved dangerously wrong	Prediction of risk and dose range from animal studies may prove unreliable: extra caution with wider margins of safety are required with ‘potentially risky modes of action’
Dosing interval between subjects	No ‘proper interval’ allowing for the observation of possible side effects was left between the dosing of one subject and the next	In fisrt-in-man studies, investigators should expect the unexpected
Preparation for adverse events	Preparation for possible adverse events (cytokine storm) was inadequate – investigators did not expect it, recognize it or treat early.	Where there is a known theoretical risk, investigators should plan for its potential occurrence

Adapted from Dayan and Wraith[8]

After this unexpected outcome of the trial United Kingdom’s Medicines and Healthcare Products Regulatory Agency (MHRA) initiated an investigation on the trial procedures and ethics. They did not find any flaw in trial procedure or in manufacture of drug. They mentioned that the severe reactions were as a result of unexpected biological effect of the drug.[9] Deficiencies they found in the drug trail were inadequate maintenance medical records, physician with inappropriate qualification, inadequacy in ensuring insurance protection of the sponsor, and failure in arranging early medical coverage.[10] In addition, there was no citation mentioned in the investigation brochure supporting the 100% homology of CD28 receptor between primate species used for preclinical trial and humans. Later, it was reviewed by Hansen and Leslie[11] that differences of up to 4% existed in the amino-acid sequences of the C”D loop of CD28 receptor in rhesus and cynomolgus with that of humans. This raises doubts on whether trial met the criteria on scientific validity of preclinical data.[10] Later, *British Journal of Medicine* and other journals requested for a more critical trial inquiry independent of the authorities who approved the trial. Toward this end, expert scientific group under Professor Gordon Duff was formed which further investigated the biological and ethical concerns which may have resulted in the disastrous aftermath.[12] Just after few minutes of drug infusion, all six human volunteers started suffering severe cytokine release syndrome leading to sever inflammation.[13] Similar effects were observed in small number of patients treated with rituximab, muromonab-CD3, and alemtuzumab antibodies.[13] Even the investigation brochure had in its text mentioned caution about possibility of cytokine release syndrome. Despite of knowing these facts infusion of TGN1412 given to all six volunteers within a short span of time was a serious concern in conduct of the trial. Moreover, when the last volunteer was to be infused, the first volunteer had already started showing adverse effects. Despite of this observation, sixth volunteer was still infused with the drug.[4] Moreover, the place where trial was conducted was not a hospital but a privately leased unit by Paraxel, which delayed the quick diagnosis and treatment of affected volunteers.[4,10] In addition, the preclinical test did not include a test for allergy. This was important because CD28 is also expressed by the cells responsible for allergy and the fact that the adverse reactions were immediate, relates to the release of preformed cytokines in granules of allergy-mediating immune cells. Inclusion of an allergy test in preclinical studies might have predicted the massive cytokine release.[14]

In an another clinical trial conducted by National Institute of Health for the drug Fialuridine, a thymidine analog having antiviral activity against Hepatitis B virus showed adverse reactions in phase 2 clinical trials leading to death of five human volunteers due to severe hepatic toxicity and lactic acidosis.[15] Before conducting human trials, Fialuridine was tested on different animals including mice, rat, dog, monkeys, and woodchucks. These studies demonstrated that doses hundred times higher than that administered to humans did not induce any toxic reactions. Moreover, animal models showed bone marrow and heart toxicity with no signs of mitochondrial injury.[16] None of the preclinical toxicity studies on laboratory animals could predict the toxic outcomes observed in phase II studies. Even a pilot study on 43 patients treated for 2 and 4 weeks duration with Fialuridine did not reveal any signs of hepatic toxicity on initial examination.[17] During 13^th^ week of phase II studies, one of the patients suddenly developed hepatic toxicity and lactic acidosis. At this point, trial was stopped for all other patients. Even after discontinuation of Fialuridine administration, seven other patients showed signs of severe hepatic toxicity five of which could not survive and other two could survive only after liver transplantation.[17] It was reported by Richardson *et al*.[18] that Fialuridine accumulates in genomic DNA in liver and also other tissues after chronic oral drug administration. Accumulation of Fialuridine in genomic DNA specifically in liver may be responsible for its toxic effects due to production of defective mitochondrial DNA resulting in high levels of lactic acid and deposition of fat in mitochondrial microvesicles.[19] Fialuridine can get incorporated into cellular and mitochondrial nacent DNA which may result in inhibition of DNA synthesis or synthesis of abnormal DNA.[17,18] This unexpected tragedy has focused attention of researchers on possibility of a new type of delayed toxic effect due to mitochondrial injury.[19] Later after these disastrous trial outcomes, woodchucks with Hepatitis B virus infection were used for evaluation of hepatotoxicity. The main aim of this study was to develop a suitable animal model, which can predict clinical toxicity in humans by preclinical studies prior to use of nucleoside analogs in clinical trials. Initial 8-week treatment showed lowering in serum levels of Hepatitis B virus but later after from 12^th^ week onward woodchucks began to loose weight and began to show mitochondrial injury.[20] Since no such chronic administration studies were carried out prior to Fialuridine clinical trial, observed chronic toxicity effects remained unpredicted by preclinical studies. Now for all preclinical studies involving nucleoside analogs for HBV treatment, a woodchuck model is used for evaluation of mitochondrial toxicity.[16]

## CONCLUSION

Drugs showing safety and efficacy in preclinical animal models may show very different pharmacological properties when administered to humans. Development of proper preclinical models which can efficiently predict drug behavior in humans is very essential prior to testing a drug in a human subject. First in man, human trials of potent biological molecules should include initial testing on very less number of human volunteers before administration of drug to a greater number of human volunteers. The above-mentioned incidents especially the TeGenero incident was an alarming call for the researchers and also for the trial approving regulatory authorities on toxicity-related unpredictability of new drugs in human subjects especially for biological with a novel mechanism of action like TGN1412. Though there is always a risk involved with clinical trials, these risks can be potentially reduced if more scientific research toward development of animal models closely mimicking drug behavior in humans can be developed.
